# An appraisal of RECQ1 expression in cancer progression

**DOI:** 10.3389/fgene.2014.00426

**Published:** 2014-12-05

**Authors:** Sudha Sharma

**Affiliations:** Department of Biochemistry and Molecular Biology, College of Medicine, Howard University, Washington, DC, USA

**Keywords:** RecQ, helicase, gene expression, vimentin, metastasis, epithelial mesenchymal transition

## Abstract

RECQ1 is the most abundant member of the human RecQ family of DNA helicases genetically linked with cancer predisposition syndromes and well known for their functions in genome stability maintenance through DNA repair. Despite being the first discovered RecQ homolog in humans, biological functions of RECQ1 have remained largely underappreciated and its relevance to cellular transformation is yet unclear. RECQ1 is overexpressed and amplified in many clinical cancer samples. *In silico* evaluation of RECQ1 mRNA expression across the NCI-60 cancer cell lines predicts an association of RECQ1 with cancer cell migration, invasion, and metastasis. Consistent with this, latest work implicates RECQ1 in regulation of gene expression, especially of those associated with cancer progression. Functionally, silencing RECQ1 expression significantly reduces cell proliferation, migration, and invasion. Collectively, these results propose that discerning the role of RECQ1 in conferring proliferative and invasive phenotype to cancer cells could be useful in developing therapeutic strategies to block primary tumor progression and metastasis.

## INTRODUCTION

The RecQ helicase family is a group of highly conserved DNA unwinding enzymes described as caretakers of the genome (Hickson, 2003; Brosh, 2013; Croteau et al., 2014). In humans, RECQ1, also known as RECQL or RECQL1, is the most abundant member of the RecQ helicase family represented by five homologs: RECQ1, WRN, BLM, RECQL4, and RECQL5 (Sharma and Brosh, 2008). Loss of function of three of the five members of the RecQ family are genetically linked with rare cancer susceptibility syndromes (Werner syndrome, Bloom syndrome, and Rothmund–Thomson syndrome) but the association of RECQ1 with cellular transformation is yet unclear. The five human RecQ helicases are expected to exhibit some functional redundancy, but the fact that the functional loss of one homolog cannot be substituted by others clearly indicates critical specialized functions.

RECQ1 is an integral component of replication complex in unperturbed cells and implicated in maintaining replication fork progression (Thangavel et al., 2010). Mechanistically, RECQ1 helicase binds and preferentially unwinds structural intermediates of DNA replication and repair, such as forked duplexes, D-loops, and Holliday junctions; and also exhibits an intrinsic ability to promote annealing of complementary single strand DNA (Sharma et al., 2005; Popuri et al., 2008). RECQ1-deficient cells are characterized by spontaneously elevated sister chromatid exchanges (Sharma and Brosh, 2008) that represent non-productive attempts to restart replication (Helleday, 2003). Furthermore, RECQ1-deficient cells accumulate DNA damage, display increased sensitivity to DNA damaging agents that induce stalled and collapsed replication forks, and exhibit chromosomal instability (Sharma and Brosh, 2007, 2008; Sharma et al., 2007). Thus it is believed that RECQ1 acts to restore productive DNA replication following stress and prevents subsequent genomic instability (Sharma and Brosh, 2008; Popuri et al., 2012; Berti et al., 2013; Sami and Sharma, 2013). RECQ1-deficiency is tolerated by cells to repair I-Sce induced double strand breaks by homologous recombination (Sharma et al., 2012), but a role of RECQ1 in replication stress induced recombination is possible given the demonstrated activity of RECQ1 protein to dissociate native Rad51-bound D-loops by branch migration (Branzei and Foiani, 2007; Bugreev et al., 2008).

Human RECQ1 is ubiquitously expressed in all cell types regardless of cell cycle stage (Kawabe et al., 2000). Previous studies have shown that RECQ1 is upregulated in rapidly dividing cells and its expression is higher in many cancer cell lines as compared to normal cells (Kawabe et al., 2000; Futami et al., 2008a). A meta-analyses of gene expression pattern included RECQ1 among the common signature genes for cancer as defined by the Gene Ontology Consortium (Xu et al., 2007). We noted that RECQ1 is overexpressed and amplified in many clinical cancer samples^1^. Forty three out of 447 differential expression analyses included RECQ1 in the top 10% upregulation list while only two did in the top 10% downregulation list in cancer versus normal^2^. Indeed, overexpression of RECQ1 has been experimentally demonstrated in human glioblastoma (Mendoza-Maldonado et al., 2011), ovarian cancer (Sanada et al., 2013), and head and neck squamous cell carcinoma (Arai et al., 2011). Furthermore, RECQ1 protein levels correlated with histological grade and Ki-67 labeling index in hepatocellular carcinoma (Futami et al., 2010) and high proliferative potential in ovarian cancer (Sanada et al., 2013). A polymorphism in RECQ1, A159C, has been associated with faster tumor progression and significantly reduced survival of pancreatic adenocarcinoma patients that received gemcitabine and radiotherapy (Li et al., 2006; Cotton et al., 2009). The A159C SNP is located in the 3^′^UTR and, thus, may functionally alter RECQ1 expression and affect clinical outcome. Remarkably, RECQ1 silencing significantly reduced proliferation of cancer cells as compared to normal cells in cell culture models and also suppressed tumor growth in mouse *xenograft* models (Sharma and Brosh, 2007; Futami et al., 2008a,b, 2010; Arai et al., 2011; Mendoza-Maldonado et al., 2011). Tumor cell growth was significantly inhibited *in vitro* by silencing RECQ1 in hypopharyngeal carcinoma cells and the combination treatment of RECQ1 siRNA and *cis*-platinum (II) diammine dichloride significantly augmented the *in vivo* anticancer effects of the drug (Arai et al., 2011). Dysfunctional checkpoint status, e.g., p53 in cancer cells may be linked to mitotic catastrophe following RECQ1 silencing (Futami et al., 2008a) but the ability of RECQ1 to facilitate recovery from replication stress could also be especially important for cancer cells (Popuri et al., 2012; Berti et al., 2013; Lu et al., 2013). Although these studies imply an association of RECQ1 with tumor growth, progression or differentiation, the molecular mechanisms through which RECQ1 might support malignant progression are not understood.

Recent analysis of genome-wide changes in gene expression has revealed a novel involvement of RECQ1 in regulation of gene expression in addition to its role in DNA damage repair (Li et al., 2014). Besides cell proliferation, the top 10 over-represented processes also included cellular movement and cell morphology suggesting that RECQ1 enhances the expression of multiple genes that play key roles in cell migration, invasion, and metastasis, including EZR, ITGA2, ITGA3, ITGB4, SMAD3, and TGFBR2. Functionally, silencing RECQ1 significantly reduced migration and invasion of the highly invasive MDA-MB-231 breast cancer cells indicating that RECQ1 plays a role in enhancing cell migration and invasion (Li et al., 2014). Consistent with the results from *in vitro* cell culture based study, high RECQ1 expression significantly associated (P = 4.2E-06) with poor overall survival in breast cancer in TCGA dataset (Gyorffy et al., 2010). High expression of RECQL4 was also associated with poor survival in breast cancer but the expression levels of WRN, BLM, and RECQL5 did not correlate with survival (Li et al., 2014).

A putative role in regulation of gene expression has been suggested by studies in *Neurospora* where a RECQ1 homolog mediates posttranscriptional gene silencing (Cogoni and Macino, 1999). Remarkably, rat RECQ1 was identified in a piRNA protein complex important for gene silencing (Lau et al., 2006), raising the possibility that human RECQ1 may function in a conserved mechanism. Although the mechanism of gene regulation by RECQ1 is yet unknown, the promoters of genes downregulated upon RECQ1 silencing were significantly enriched for a potential G4 sequence motif which are associated with potential to form G-quadruplex structures in DNA (G4 DNA; Li et al., 2014). Chromatin immunoprecipitation experiments demonstrated RECQ1 binding to G4 sequence motifs in the promoters of select downregulated genes indicating that RECQ1 may modulate gene expression by regulating the *in vivo* stability of G4 DNA structures. Elegant studies from the Maizels (Gray et al., 2014), Monnat and Harris (Nguyen et al., 2014) labs have recently identified that gene regulatory functions of XPB, XPD, and BLM helicases also involve G4 DNA suggesting that the binding to G4 motifs may be a common mechanism of transcriptional regulation by these distinct DNA helicases. Importantly, similar to XPB, but unlike BLM, RECQ1 binds but does not unwind G4 DNA *in vitro* (Popuri et al., 2008; Sharma, 2011). It is likely that binding to specific G4 sequence motifs by each of these helicases may characterize specific signaling and regulatory pathways associated with cancer. In addition to gene regulatory regions, G4 sequence motifs are also present at chromatin regions preferentially bound by RECQ1 such as replication origins, fragile sites, and telomeres indicating that RECQ1 functions are especially critical at genomic loci having potential to form secondary structures (Thangavel et al., 2010; Lu et al., 2013; Maizels and Gray, 2013; Popuri et al., 2014).

## RECQ1 EXPRESSION ACROSS THE NCI-60 HUMAN TUMOR CELL LINES

**FIGURE 1:**
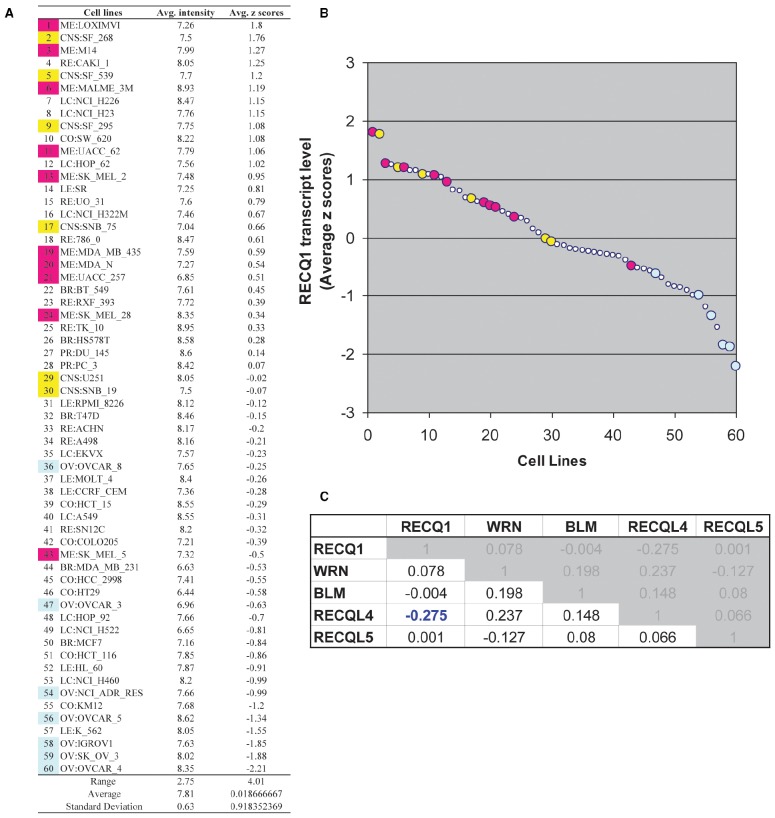
**RECQ1 transcript levels in the NCI-60 human tumor cell lines. (A)** Table of average intensity (determined from log 2 intensity values from Affymetrix microarrays) and their combined *z*-score means presented in descending order. **(B)** Scatter plot depicting the distribution of RECQ1 transcript levels across the NCI-60. The numerical series (1-to-60) for the cell lines from panel **(A)** correspond to the *x*-axis. Cell lines from the central nervous system (shown in yellow) and melanoma (shown in pink) displayed higher transcript levels of RECQ1, while ovarian cancer cell lines (shown in blue) displayed the least. **(C)** Cross-correlation among RecQ family. Pearson’s correlation coefficients between transcripts of the known five RecQ homologs in the NCI-60. Only significant correlation of RECQ1 in the NCI-60 is with the RECQL4 transcripts. Processing and normalization of transcript expression data from the NCI-60 has been described previously (Reinhold et al., 2012). Normalization of Affymetrix HG-U95, HG-U133, HG-U133 plus 2.0 and Affy HuEx 1.0 is done by GC robust multi-*array* average (GCRMA). Data is accessible at http://discover.nci.nih.gov by using RECQL (RECQ1) as input in Cellminer.

By virtue of its overexpression in cancer and its association with cell proliferation, migration, and invasion, RECQ1 can potentially serve as a predictive biomarker and attractive target for cancer therapy. To assess gene expression pattern of RECQ1 in various tumors, we utilized a panel of 60 human tumor cell lines derived from nine different tissues of origin (NCI-60) that has been extensively characterized for gene expression and copy-number variations, and commonly used for genetic analysis and screening of potential chemotherapeutic agents (Shoemaker, 2006; Weinstein, 2006; Liu et al., 2010). We conducted *in silico* analysis of RECQ1 expression pattern across the NCI-60 employing a publicly accessible webtool CellMiner^3^ (Reinhold et al., 2012; Figure 1). RECQ1 mRNA expression in the NCI-60 panel is shown in Figure 1A both as average transcript intensity values and as the average *z* score that allows a better comparison of relative expression. Noticeably, the NCI-60 cell lines exhibit a wide spectrum of RECQ1 expression with transcript level values ranging from both lower and higher than the mean (Figure 1B). Cell lines melanoma LOXIMVI and central nervous system SF_268 showed the highest RECQ1 mRNA levels, whereas ovarian cancer cell lines OVCAR_4 and SK_OV_3 showed the lowest (Figure 1B). Nine out of the 10 melanoma cell lines and 4 out of 6 central nervous system cell lines expressed high RECQ1 mRNA (Figure 1B). SK_MEL_5 cells were the only melanoma cells within the NCI-60 panel with lower RECQ1 transcript levels. The lung and renal cell lines were most variable in RECQ1 expression and the ovarian cell lines expressed lowest levels of RECQ1 transcript. It remains to be tested whether RECQ1 transcript levels correlate with the protein level, but a statistically significant correlation with DNA copy number (Pearson’s correlation *R* = 0.38; *P* = 0.003) was observed with the estimated RECQ1 DNA copy number ranging from 3.67 for lung cancer cell line NCI_H322M to 1.35 in ovarian cancer cell line SK_OV_3. Expression profiles of five known RecQ family genes across the NCI-60 panel revealed no significant correlation of RECQ1 mRNA expression with WRN, BLM, or RECQL5; however, RECQ1 expression displayed significant inverse correlation with RECQL4 (Figure 1C).

**FIGURE 2:**
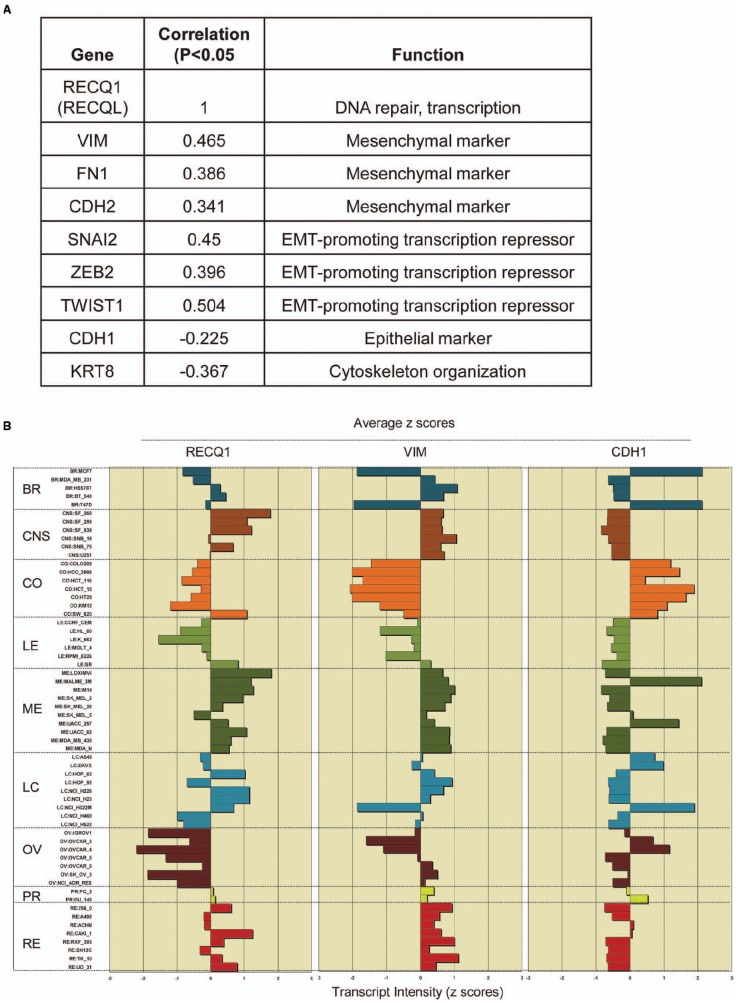
**RECQ1 expression in the NCI-60 panel correlates with markers of tumor progression. (A)** Significant correlations of RECQ1 with select genes involved the cell invasion, migration, and metastasis. The numbers in the table are expression profile correlations for expression of gene pairs in the NCI-60 panel. EMT, epithelial to mesenchymal transition; **(B)** RECQ1 expression is highly correlated with the mesenchymal marker vimentin. Comparison of expression profiles for RECQ1, VIM (vimentin), and CDH1 (E-cadherin) across the NCI-60 cell lines. Mean-centered transcript *z*-cores are plotted on the *x*-axis; bars to the right show increased expression, bars to the left show decreased expression relative to the expression mean. The cell lines on the *y*-axis are grouped by tissue of origin. BR, breast; CNS, central nervous system; CO, colon; LE, leukemia; ME, melanoma; LC, lung cancer; OV, ovarian; PR, prostrate; RE, renal. Data was generated querying RECQL (RECQ1) as input in Cellminer (http://discover.nci.nih.gov/cellminer/).

Recent studies using the NCI-60 panel have shown that the genes whose expression at the mRNA level is correlated over diverse cell lines are likely to function together in a molecular network regulating these processes (Kohn et al., 2012, 2014). We postulated that the ability of RECQ1 to promote tumor cell migration and invasion involves changes in expression of specific genes that are critical to these processes. Using the CellMiner NCI-60 analysis tools, we searched for genes that were significantly correlated with RECQ1-expression across the NCI-60 panel and had established functions in cell migration and invasion (Figure 2A). Notably, RECQ1 expression displayed significant positive correlation with mesenchymal differentiation markers vimentin, N-cadherin, and fibronectin (VIM, FN1, and CDH2), and transcription factors that promote these processes (TWIST, SNAI2, and ZEB2). Furthermore, RECQ1 expression correlated significantly and negatively with genes maintaining cellular structural integrity (KRT8) and epithelial marker E-cadherin (CDH1). Correlation of the mRNA transcript levels of RECQ1 with VIM and CDH1 across the NCI-60 tumor cell lines is depicted in Figure 2B. The NCI-60 gene expression profile for CDH1 is inversely correlated to that of the gene VIM which is considered as a hallmark of mesenchymal-like conversion of epithelial cells in carcinomas (Zeisberg and Neilson, 2009; Kohn et al., 2014).

## PREDICTIVE AND PROGNOSTIC POTENTIAL OF RECQ1 EXPRESSION IN CANCER

Predictions from *in silico* analysis across the NCI-60 panel support previously reported overexpression of RECQ1 in various cancers and indicate RECQ1 expression to be especially significant in tumors of central nervous system origin and melanoma. Evidently, RECQ1 is highly expressed in human brain glioblastoma relative to control brain tissues and its depletion affects proliferation of glioblastoma cells and causes an increased level of DNA damages (Mendoza-Maldonado et al., 2011). Examining RECQ1 gene expression using the ONCOMINE mRNA microarray database^4^ revealed that RECQ1 is significantly upregulated in brain and central nervous system tumors, including glioblastoma, when compared to normal tissue. Overexpression of DNA repair genes is associated with metastasis in melanomas (Kauffmann et al., 2008), and the expression levels of RECQ1 significantly correlated with DNA repair genes displaying functional network which are commonly overexpressed in tumors with poorer prognosis in melanoma (Jewell et al., 2010). Given the demonstrated roles of RECQ1 in repairing DNA damage caused by chemotherapeutic agents such as ionizing radiation, camptothecin, and temozolomide, overexpression of RECQ1 may provide a survival advantage to melanoma cells by promoting the ability of cancer cells to tolerate genotoxic stress. In contrast, WRN and RECQL4 may have a tumor suppressor function in melanoma since melanoma has been reported in patients with loss of function mutations in these RecQ proteins (Howell and Bray, 2008; Monnat, 2010). Notably, within the RecQ family, RECQ1 expression correlated significantly and negatively with RECQL4 across the NCI-60 panel. Lao et al. (2013) have recently shown that the expression of BLM and RECQL4 is increased in primary colorectal cancer whereas expression levels of RECQ1 and RECQL5 are decreased. Consistent with the observation in primary colorectal cancer patient samples, low expression of RECQ1 and significantly high expression of RECQL4 were observed in colorectal cancer cell lines as compared to normal colonic mucosa indicating the feasibility of using cell lines to study the functional consequences of alterations in the expression levels of the RecQ helicases (Lao et al., 2013).

RecQ helicases contribute multiple biochemical activities to various DNA repair processes and loss of their functions leads to increased DNA damage, genomic instability, and enhanced sensitivity to certain chemotherapeutic agents (Brosh, 2013). Therefore, altered expression of RecQ helicases may be useful in predicting patient’s response to these DNA damaging drugs used for treatment of cancer. Indeed RECQ1 expression correlated with cisplatin resistance in oral squamous cell carcinoma (Zhang et al., 2006) and the depletion of RECQ1 significantly augmented the *in vivo* anticancer effects of the drug cis-platinum (II) diammine dichloride (Arai et al., 2011). This is consistent with the fact that cisplatin and related platinum drugs induce inter-strand cross links in DNA that impair progression of replication forks and RECQ1 helicase functions are important to restore productive DNA replication (Sami and Sharma, 2013). However, RECQ1 expression was found to be elevated in ovarian cancer cells that were sensitive to carboplatin as compared to those which were carboplatin-resistant (Peters et al., 2005) indicating complex mechanisms of platinum resistance (Stewart, 2007). Thus, a better correlation of RECQ1 expression with the molecular characteristics and heterogeneity of cancer needs to be established in order to test the use of RECQ1 as a potential biomarker.

In addition to unlimited proliferative potential, a tumor cell must acquire the ability to migrate and invade normal tissues to become fully malignant (Hanahan and Weinberg, 2011). Invasion of tumor cells and metastatic spread to distant organs relies on complex molecular interactions including diminished epithelial characteristics and enhanced mesenchymal attributes (Kalluri and Weinberg, 2009). RECQ1 expression across the NCI-60 panel significantly correlated with a loss of the epithelial marker CDH1 and acquisition of expression of the mesenchymal markers including VIM. Correlation with VIM in the NCI-60 panel raises the question whether RECQ1 expression is associated with the epithelial to mesenchymal transition (EMT) which is among the central mechanisms to induce invasiveness and metastasis of tumors. Indeed, RECQ1-depletion decreased cell migration and invasion in cervical adenocarcinoma HeLa and breast cancer MDA-MB-231 cell lines (Li et al., 2014). Similarly, RECQ1 silencing in oral squamous cell carcinoma SCC-9 cells downregulated the expression level of immunosuppressive factors that are necessary for regulating the migration of tumor cells (Tao et al., 2014). Collectively these results propose that RECQ1 may contribute to tumor progression by regulating key genes that promote cancer cell migration, invasion, and metastasis. It is plausible that similar to what has been shown for a few other proteins involved in DNA damage response and repair, RECQ1 participates in transcription regulation either by binding directly to DNA or through interaction with specific transcription factors (Featherstone and Jackson, 1999; Mullan et al., 2006; Kraus, 2008; Jaehnig et al., 2013; Broustas and Lieberman, 2014). A systematic investigation of RECQ1-regulated transcriptome may uncover the gene networks regulated by RECQ1 in the context of cancer progression.

Increased expression of several DNA repair proteins has been correlated with cellular invasiveness in cancer (Mitra et al., 2009; Barbano et al., 2011; Martinez-Marignac et al., 2011; Yuan et al., 2012); however, little effort has been made thus far to determine any role of RecQ helicase homologs in tumor progression and metastasis. Understanding the role of RECQ1 in conferring proliferative and invasive phenotype to cancer cells could be useful in developing therapeutic strategies to block primary tumor progression and metastases. Functional evidence from RECQ1 silencing, and the projected correlation with VIM expression in the NCI-60 panel implies an association of RECQ1 with more aggressive disease (Zeisberg and Neilson, 2009). Future studies should examine whether RECQ1 mRNA and/or protein levels can predict invasion and metastatic potential of a tumor. Predictions from large human cancer cell line panels that capture tumor heterogeneity, such as NCI-60, and the availability of clinically and molecularly annotated patient tumor datasets for multiple tumor types, such as TCGA, should provide additional opportunities to test the significance of RECQ1 in developing new *in vitro* preclinical models for cancer detection and treatment.

### Conflict of Interest Statement

The author declares that the research was conducted in the absence of any commercial or financial relationships that could be construed as a potential conflict of interest.

## Footnotes

1 www.cbioportal.org

2 www.oncomine.org

3 http://discover.nci.nih.gov/cellminer/

4 www.oncomine.org
